# Systematic metabolic profiling and bioactivity assays for bioconversion of Aceraceae family

**DOI:** 10.1371/journal.pone.0198739

**Published:** 2018-06-07

**Authors:** Jinyong Park, Dong Ho Suh, Digar Singh, Sarah Lee, Jong Seok Lee, Choong Hwan Lee

**Affiliations:** 1 Department of Bioscience and Biotechnology, Konkuk University, Seoul, Republic of Korea; 2 National Institute of Biological Resources, Environmental Research Complex, Incheon, Republic of Korea; Korea University, REPUBLIC OF KOREA

## Abstract

Plants are an important and inexhaustible source of bioactive molecules in food, medicine, agriculture, and industry. In this study, we performed systematic liquid chromatography–mass spectrometry (LC-MS)-based metabolic profiling coupled with antioxidant assays for indigenous plant family extracts. Partial least-squares discriminant analysis of LC-MS datasets for the extracts of 34 plant species belonging to the families Aceraceae, Asteraceae, and Rosaceae showed that these species were clustered according to their respective phylogenies. In particular, seven Aceraceae species were clearly demarcated with higher average antioxidant activities, rationalizing their application for bioconversion studies. On the basis of further evaluation of the interspecies variability of metabolic profiles and antioxidant activities among Aceraceae family plants, we found that *Acer tataricum* (TA) extracts were clearly distinguished from those of other species, with a higher relative abundance of tannin derivatives. Further, we detected a strong positive correlation between most tannin derivatives and the observed higher antioxidant activities. Following *Aspergillus oryzae*-mediated fermentative bioconversion of *Acer* plant extracts, we observed a time-correlated (0–8 days) linear increase in antioxidant phenotypes for all species, with TA having the highest activity. Temporal analysis of the MS data revealed tannin bioconversion mechanisms with a relatively higher abundance of gallic acid (m/z 169) accumulated at the end of 8 days, particularly in TA. Similarly, quercetin precursor (glycoside) metabolites were also transformed to quercetin aglycones (m/z 301) in most *Acer* plant extracts. The present study underscores the efficacy of fermentative bioconversion strategies aimed at enhancing the quality and availability of bioactive metabolites from plant extracts.

## Introduction

Bioconversion of renewable plant biomass with the aim of tapping their high-end value-added pool of molecules for use as bioactives and food additives and inclusion in cosmetics and pharmaceuticals has attracted considerable attention in recent years. In general, this myriad of valuable plant-derived molecules includes antibiotics, alkaloids, antimicrobials, food-grade pigments, and phenolics [1]. In particular, plant-derived polyphenols and flavonoids have numerous health-related effects, including antioxidant, anticancer, and anti-obesity activities [2]. Hence, the inclusion of plant-derived functional bioactives as dietary supplements has increasingly been realized owing to their higher efficacies coupled with lower toxicity compared with conventional medicines [3]. Ushered by the soaring demands for plant-derived bioactive ingredients, microbial biotransformation and biovalorization of agro-waste and plant biomass through microbial fermentation have emerged as sustainable and environment-friendly alternatives. Given that plant biomass is a potentially inexhaustible source of valuable products, the enhanced production and bioconversion of natural products, e.g., bioactive secondary metabolites, using microbial fermentation have gained widespread interest [4, 5].

Among angiosperms, plants belonging to the families Aceraceae, Asteraceae, and Rosaceae have virtually ubiquitous distribution, and are renowned for their food, herbal medicine, and ornamental applications. The Aceraceae (maple) family comprises 129 species, most of which are trees and shrubs distributed widely in temperate regions of the northern hemisphere, including North-Eastern America, Europe, and East Asia [6]. Aceraceae contains two commercially important genera, i.e., *Diptesonia* and *Acer*, the latter of which has been harnessed for commercial purposes, such as the preparation of traditional medicines in East Asia (owing to their high antioxidant, antitumor, and anti-inflammatory activities) and production of Maple syrup in the West [7]. Plants in the Asteraceae (daisy) family, of which there are over 25,000 species, have traditionally been used as sources of rubber, edible oils, and vegetables with associated antioxidant functions [8]. Quintessentially, species of Asteraceae contain a high abundance of sesquiterpenoids that have a myriad of pharmacological applications as growth regulators and antifungal and antitumor compounds [9]. Similarly, the Rosaceae family represents another dominant group of flowering plants containing an abundance of bioactive metabolites with antioxidant, estrogenic, and anti-proliferative activities [10].

Recently, the fermentative bioconversion of agro-industrial and plant biomass has attracted renewed interest with a view toward exploiting the bioavailability of valuable natural products. Analogous to food fermentative bioprocesses, in which secretion of hydrolytic enzymes promotes the release of flavor and functional metabolites, the fermentative bioconversion of plant biomass or its crude extracts typically produces valuable phytochemicals from plant matrices [11, 12]. Previously, a number of studies have highlighted the bioconversion and biovalorization of agro-industrial products, including capsaicin hydrolysates [13], kaempferol from cauliflower outer leaves [14], soy isoflavones in soy extracts [15], and pine polyphenols [16]. Since most of these studies have focused on selected groups of molecules or their targeted bioconversion, we conjecture that a comprehensive high-throughput and high-fidelity MS-analysis of the intermediate metabolic entities would make a valuable contribution toward attaining the mechanistic understanding if the bioconversion processes. Under current perspectives, metabolomic analysis, which can provide an unbiased snapshot of the quantitative and quantitative trends of low molecular weight metabolites, can be used to generate a holistic picture of the overall metabolic events concomitant with a bioconversion event.

In this study, we performed comprehensive metabolite profiling of different plant species (34) belonging to three commercially important plant families (Aceraceae, Asteraceae, and Rosaceae) abundant throughout the Korean Peninsula. The plant extracts with significantly higher antioxidant activities were screened and subjected to fermentative bioconversion using *Aspergillus oryzae* to examine time-correlated alterations in corresponding antioxidant potentials.

## Materials and methods

### Chemicals and reagents

Analytical grade ethanol, methanol, acetonitrile, and water were purchased from Fisher Scientific (Pittsburgh, PA, USA). 2,2'-Azino-bis (3-ethylbenzothiazoline-6-sulfonic acid) diammonium salt (ABTS), 2,2-diphenyl-1-picrylhydrazyl (DPPH), 6-hydroxy-2,5,7,8-tetramethylchromane-2-carboxylic acid (Trolox), 2,4,6-Tris(2-pyridyl)-s-triazine (TPTZ), acetic acid, formic acid, glycerol, iron (III) chloride, potassium persulfate, sodium hydroxide, sodium acetate, and standard compounds were obtained from Sigma-Aldrich (St. Louis, MO).

### Plant materials

Overall, 34 native Korean plant species from the families Aceraceae (7), Asteraceae (9), and Rosaceae (18) were examined in this study. The plant samples were procured from the National Institute of Biological Resources (NIBR, Incheon, Republic of Korea). Detailed information on the respective plant species, including their harvest zones and collection dates, and NIBR sample accession numbers is listed in Table 1. The plant samples were collected between May and October 2014 from two megalopolises, seven provinces, and one special self-governing province in the Republic of Korea. The field studies by NIBR and harvested samples did not involve any endangered or protected species.

**Table 1:** Information on the 34 native Korean plant species used in this study.

No.	Family	Genus	Species	Collection Area	Collection Date	NIBR number
1	Aceraceae	Acer	triflorum	Sangjung-ri, Geumgwang-myeon, Anseong-si, Gyeonggi-do	2014-07-25	NIBR2014-23
2		Acer	pictumsubsp.mono	Sangjung-ri, Geumgwang-myeon, Anseong-si, Gyeonggi-do	2014-07-25	NIBR2014-27
3		Acer	buergerianum	Janghyeon-ri, Cheongna-myeon, Boryeong-si, Chungcheongnam-do	2014-08-07	NIBR2014-53
4		Acer	komarovii	Gohan-ri, Gohan-eup, Jeongseon-gun, Gangwon-do	2014-08-30	NIBR2014-144
5		Acer	tataricum	Gurae-ri, Sangdong-eup, Yeongwol-gun, Gangwon-do	2014-08-30	NIBR2014-148
6		Acer	pseudosieboldianum	Gohan-ri, Gohan-eup, Jeongseon-gun, Gangwon-do	2014-08-30	NIBR2014-150
7		Acer	pictum	Jeodong-ri, Ulleung-eup, Ulleung-gun, Gyeongsangbuk-do	2014-07-16	NIBR2014-155
8	Asteraceae	Artemisia	capillaris	Nadae-ri, Yaro-myeon, Hapcheon-gun, Gyeongsangnam-do	2014-08-21	NIBR2014-98
9		Aster	pinnatifidus	Geogi-ri, Jusang-myeon, Geochang-gun, Gyeongsangnam-do	2014-08-22	NIBR2014-115
10		Bidens	bipinnata	Dongmak-ri, Yeoncheon-eup, Yeoncheon-gun, Gyeonggi-do	2014-08-22	NIBR2014-173
11		Conyza	canadensis	Sangdodae-ri, Sangchon-myeon, Yeongdong-gun, Chungcheongbuk-do	2014-08-14	NIBR2014-79
12		Erigeron	annuus	Dongmak-ri, Yeoncheon-eup, Yeoncheon-gun, Gyeonggi-do	2014-08-14	NIBR2014-163
13		Helianthus	tuberosus	Jiro-ri, Byeongyeong-myeon, Gangjin-gun, Jeollanam-do	2014-08-12	NIBR2014-67
14		Lactuca	indica	Gomo-ri, Soheul-eup, Pocheon-si, Gyeonggi-do	2014-08-24	NIBR2014-182
15		Saussurea	pulchella	Gohan-ri, Gohan-eup, Jeongseon-gun, Gangwon-do	2014-08-30	NIBR2014-140
16		Sigesbeckia	pubescens	Gurae-ri, Sangdong-eup, Yeongwol-gun, Gangwon-do	2014-08-30	NIBR2014-146
17	Rosaceae	Chaenomeles	sinensis	Ojeong-dong, Daedeok-gu, Daejeon	2014-08-10	NIBR2014-62
18		Crataegus	pinnatifida	Gurae-ri, Sangdong-eup, Yeongwol-gun, Gangwon-do	2014-08-30	NIBR2014-147
19		Eriobotrya	japonica	Jiro-ri, Byeongyeong-myeon, Gangjin-gun, Jeollanam-do	2014-08-13	NIBR2014-69
20		Pourthiaea	villosa	Seonheul-ri, Jocheon-eup, Jeju-si, Jeju special self-governing province	2014-08-24	NIBR2014-120
21		Prunus	armeniaca	Ojeong-dong, Daedeok-gu, Daejeon	2014-07-20	NIBR2014-11
22		Prunus	yedoensis	Janghyeon-ri, Cheongna-myeon, Boryeong-si, Chungcheongnam-do	2014-08-07	NIBR2014-52
23		Prunus	maackii	Gurae-ri, Sangdong-eup, Yeongwol-gun, Gangwon-do	2014-08-30	NIBR2014-137
24		Prunus	padus	Gohan-ri, Gohan-eup, Jeongseon-gun, Gangwon-do	2014-05-22	NIBR2014-160
25		Prunus	prunus sp.	Gomo-ri, Soheul-eup, Pocheon-si, Gyeonggi-do	2014-08-08	NIBR2014-171
26		Pyrus	ussuriensis	Icheon-ri, Sangbuk-myeon, Ulju-gun, Ulsan	2014-08-08	NIBR2014-43
27		Rosa	multiflora	Nadae-ri, Yaro-myeon, Hapcheon-gun, Gyeongsangnam-do	2014-08-21	NIBR2014-94
28		Rubus	coreanus	Sogye-ri, Hwanggan-myeon, Yeongdong-gun, Chungcheongbuk-do	2014-08-14	NIBR2014-77
29		Rubus	crataegifolius	Nadae-ri, Yaro-myeon, Hapcheon-gun, Gyeongsangnam-do	2014-08-21	NIBR2014-103
30		Rubus	phoenicolasius	Nadae-ri, Yaro-myeon, Hapcheon-gun, Gyeongsangnam-do	2014-08-21	NIBR2014-108
31		Sanguisorba	officinalis	Nadae-ri, Yaro-myeon, Hapcheon-gun, Gyeongsangnam-do	2014-08-21	NIBR2014-110
32		Sorbus	commixta	Jeodong-ri, Ulleung-eup, Ulleung-gun, Gyeongsangbuk-do	2014-07-16	NIBR2014-6
33		Spiraea	prunifolia	Ungyo-ri, Bangnim-myeon, Pyeongchang-gun, Gangwon-do	2014-08-08	NIBR2014-56
34		Spiraea	salicifolia	Ungyo-ri, Bangnim-myeon, Pyeongchang-gun, Gangwon-do	2014-08-08	NIBR2014-58

### Sample preparation for metabolite profiling and antioxidant activity screening

Plant samples were dried under shade, and each sample (100 g) was extracted three times with 70% ethanol (1 L). The filtered sample extracts were dried using a rotary vacuum evaporator (Eyela, Tokyo, Japan), and were subsequently freeze-dried and stored under deep-freezing conditions (-70 °C) until further analyses. Each of the dried sample extracts (20 mg) was dissolved with 1 mL of 70% ethanol and filtered through a 0.2-μm polytetrafluoroethylene (PTFE) membrane prior to ultra-performance liquid chromatography–quadrupole–time of flight–mass spectrometry (UPLC-Q-TOF-MS) and ultrahigh-performance liquid chromatography–LTQ–linear ion trap–tandem mass spectrometry (UHPLC-LTQ-IT-MS/MS) analyses.

### UHPLC-LTQ-XL-IT-MS/MS analysis

The Thermo Fischer Scientific LTQ XL linear ion trap mass spectrometry system used in the present study consisted of an electrospray interface (Thermo Fischer Scientific, San José, CA, USA) coupled with a DIONEX UltiMate 3000 RS Pump, RS Autosampler, RS Column Compartment, and RS Diode Array Detector (Dionex Corporation, Sunnyvale, CA, USA). The samples were separated on a Thermo Scientific Syncronis C18 UHPLC column with 1.7 μm particle size. The mobile phase consisted of solvent A [0.1% (v/v) formic acid in water] and solvent B [0.1% (v/v) formic acid in acetonitrile]. The gradient conditions were increased from 10% to 100% solvent B over 18 min, and re-equilibrated to the initial conditions for 4 min. The flow rate was 0.3 mL/min and the injection volume was 10 μL. The temperature of the column during measurement was maintained at 35 °C. The photodiode array was set at 200–600 nm for detection and managed by 3D field. Ion trap was performed in positive, negative, and full-scan ion modes within a range of 150–1000 *m/z*. The operating parameters were as follows: source voltage, ±5 kV, capillary voltage, 39 V; and capillary temperature, 275 °C. Tandem MS analysis was performed using scan-type turbo data-dependent scanning under the same conditions as those used for MS scanning. The samples were analyzed using three biological replicates for each sample. To circumvent systematic errors during analysis, the samples were analyzed in random blocks of 10 runs, followed by an intermittent quality control (QC) sample prepared from pooled blends of each sample extract.

### UPLC-Q-TOF-MS analysis

UPLC-Q-TOF-MS analysis was performed using a Waters Micromass Q-TOF Premier mass spectrometer coupled to an UPLC Acquity system (Waters, Milford, MA) equipped with a binary solvent delivery system, an autosampler, and an ultraviolet (UV) detector. The samples were separated on an Acquity UPLC BEH C18 column (100 mm × 2.1 mm × 1.7 μm particle size; Waters Corp.). The sample injection volume was 5 μL with a mobile phase flow rate of 0.3 mL/min, and the column temperature was maintained at 37 °C. The binary mobile phase consisted of solvent A (0.1% formic acid in water) and solvent B (0.1% formic acid in acetonitrile). The mobile phase solvent gradient was programmed as follows: 5% solvent B for 1 min, gradually increased to 100% solvent B over 9 min, maintained as such for the next 1 min, and finally decreased to 5% Solvent B over 3 min, with a total run time of 13 min. The MS data were collected in the range of 100–1000 *m/z*, within negative and positive ion modes. The source temperature was 100 °C, the desolvation gas (nitrogen) was set to 600 L/h at a temperature of 200 °C, and the cone gas (nitrogen) was set to 50 L/h. The capillary voltage and cone voltage were set at 2.5 kV and 50 V, respectively. Data were collected with a scan accumulation time of 0.2 s in the centroid mode. Leucine enkephalin (10 ppm) was used as the reference lock mass [m/z 554.2615 (-) and 556.2771 (+)] at a flow rate of 10 μL/min. All samples were analyzed using three biological replicates and a random block of 10 samples with intermittent QC samples.

### Data processing and multivariate analysis

The UHPLC-LTQ-IT-MS/MS data were acquired using Xcalibur software (version 2.00; Thermo Fisher Scientific), and raw data were subsequently converted to netCDF (*.cdf) format using Xcalibur software. The MS data files were then processed using MetAlign software (RIKILT-Institute of Food Safety, Wageningen, The Netherlands) to evaluate the retention times, normalized peak intensities, and accurate masses. The resulting data were exported to an Excel file (Microsoft, Redmond, WA, USA), and multivariate statistical analyses were performed using SIMCA-P+ software (version 12.0; Umetrics, Umea, Sweden). Principal component analysis (PCA) and loading plots were employed to compare metabolic variants among the plant sample extracts. The variables were selected based on the variable importance in the projection (VIP) value, whereas significant differences between experimental groups were determined by analysis of variance (ANOVA). PASW Statistics (version 18.0; SPSS, Inc., Chicago, IL) was used to calculate Pearson correlation coefficients between metabolites and bioactivity assays as well as imputing the missing values while data processing. Heat map and correlation maps were visualized using MEV software (version 4.8; Romania).

### Bioactivity assays

A modified version of the method described by Re et al. [17] was used to perform the ABTS assay. Briefly, 7 mM ABTS was mixed in 2.45 mM potassium persulfate solution, and the mixture was stored for 12 h in the dark at room temperature. The solution was diluted with deionized water until the absorbance reached 0.7 ± 0.02 at 750 nm, as determined using a microplate reader (Spectronic Genesys 6; Thermo Electron, Madison, WI, USA). Sample extracts (10 μL) obtained from plants in each of the three plant families were mixed with 190 μL of diluted ABTS solution in the wells of a 96-well plate, and subsequently incubated at 37 °C in the dark for 6 min. Following incubation, the absorbance of the reacted samples was recorded at 750 nm using a microplate reader.

The DPPH assay was performed using the method described by Dietz et al. [18] with a few modifications. Plant extracts (20 μL) from each of the species from the three families were added to 0.2 mM DPPH ethanol solution (180 μL) in the wells of a 96-well plate, and then incubated for 20 min in the dark at room temperature. The absorbance was recorded at 515 nm using a microplate reader.

The ferric reducing ability of plasma (FRAP) assay was performed using a modified version of the procedure previously described by Benzie and Strain [19]. The FRAP reagent was mixed with 300 mM sodium acetate buffer (pH 3.6), 10 mM TPTZ, and 20 mM ferric chloride at a ratio of 10:1:1. Each plant sample (10 μL) was mixed with 300 μL of FRAP reagent in the wells of a 96-well plate and incubated in the dark at 37 °C for 6 min. The absorbance at 593 nm was then measured.

All bioactivity assays were conducted using three biological as well as analytical replicates for each sample and the results were expressed as the Trolox equivalents of antioxidant capacity, with a concentration range of 0.0156–1 mM.

### Fungal strains and inoculum preparation

The *Aspergillus oryzae* KCCM 12698 strain used in the study was procured from the Korean Culture Center of Microorganisms (KCCM, Seoul, Republic of Korea). The strain was transferred from -80 °C frozen stocks to malt extract agar (MEA) plates for pre-culture prior to use as an inoculum. The frozen stocks were grown on MEA plates for 7 days at 28 °C in the dark and thereafter sub-cultured. The culture inoculum was prepared by harvesting the fresh spores from MEA cultures using 2 mL of sterile distilled water containing 0.1% Tween 80. The inoculum was dilution adjusted to 1.8 × 10^7^ spores/mL.

### Preparation of the *Acer* mixed broth and fermentative bioconversion

*Acer* plant extracts (0.25 g) were mixed with 50 mL of MEB (0.5%, w/v) prior to autoclave sterilization. One milliliter of *A*. *oryzae* inoculum was added to each culture flask and the cultures were incubated at 28 °C for 8 days. The broth samples (3 mL) for metabolite extraction were regularly harvested at an interval of 2 days, and the aliquots were stored immediately under deep-freezing (-20 °C) conditions until analyses.

The fermented samples were extracted by adding 6 mL of 70% ethanol to each aliquot sample, followed by vigorous mixing in a rotary shaker at 200 rpm for 2 h, and then centrifuged at 5,000 rpm and 4 °C for 10 min (Universal320; Hettich Zentrifugen, Germany). The supernatants were collected and dried in a speed vacuum concentrator (Biotron, Seoul, Republic of Korea). The extracted sample analytes were re-suspended in 70% ethanol to a fixed titer of 10 mg/mL and filtered using a disposable 0.45-μm PTFE filter membrane prior to further analyses. The bioconversion steps were performed for seven *Acer* species extracts using three biological replicates for each.

## Results

### Metabolite profiling and antioxidant activity screening for three plant families

**Fig 1:**
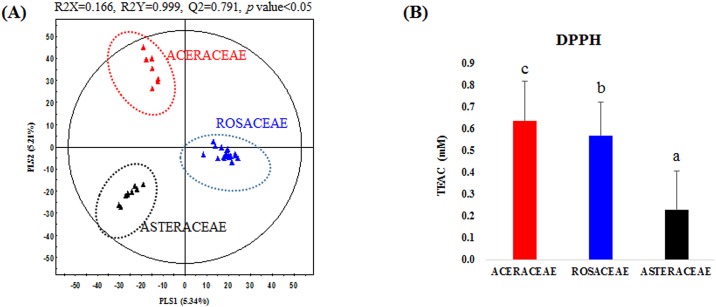
(A) Partial least-square discriminant analysis score plot based on UHPLC–LTQ-IT-MS/MS datasets, and (B) average antioxidant activity (2,2-diphenyl-1-picrylhydrazyl: DPPH), for the metabolite extracts derived from plant species belonging to the families Aceraceae, Rosaceae, and Asteraceae. The different letters are indicative of statistically significant differences for observed bioactivities according to Duncan’s multiple-range test at *p* < 0.05.

In order to select the plant material extracts for bioconversion, we initially performed metabolite profiling coupled with multivariate statistical analysis for plants in three abundant and commercially important plant families in Korea (Aceraceae, Asteraceae, and Rosaceae). The partial least-square discrimination analysis (PLS-DA) score plot presented in Fig 1A shows that the 34 native Korean plant species were clustered depending on their phylogeny. The quality parameters of PLS-DA were signified using R^2^X (0.166), R^2^Y (0.999), Q^2^ (0.791), and *p*-value < 0.05, suggesting their high predictive accuracies. In addition, the bioactivity potentials of the three plant families were evaluated through examining the antioxidant activities of the extracts (Fig 1B). The average antioxidant activity (±SD) values for sample extracts from plants in each family were expressed as the Trolox equivalent antioxidant capacity. Although, observed differences in the antioxidant activities of plant extracts among the three different plant families were not statistically significant, the DPPH assay indicated that antioxidant activities were highest for Aceraceae family plant extracts, followed by Rosaceae and Asteraceae. Henceforth, we focused our next phase of study selectively on Aceraceae family plants owing to their relatively higher antioxidant activities as well as distinct metabolite compositions.

**Fig 2:**
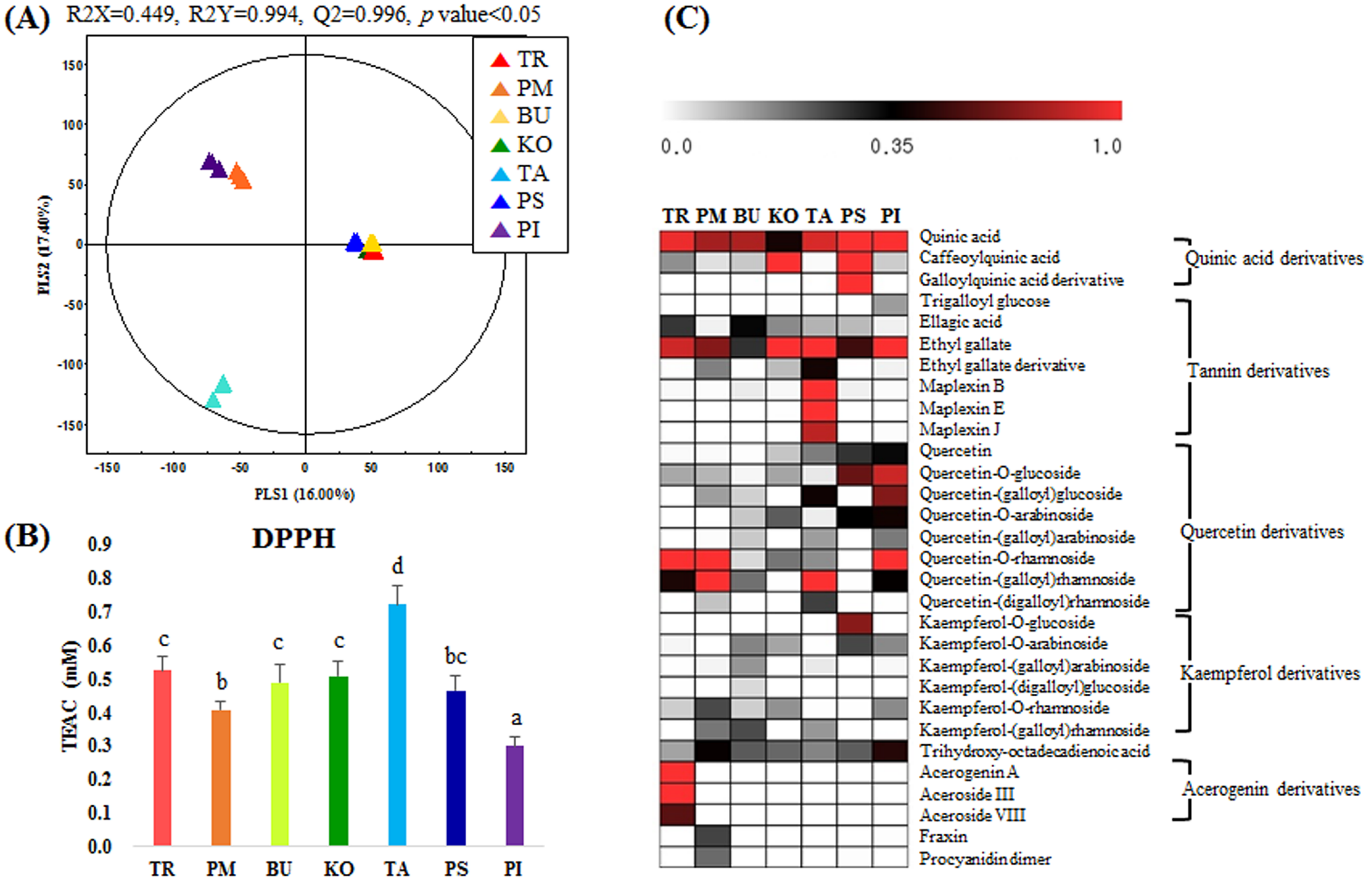
(A) Partial least-square discriminant analysis (PLS-DA) score plot based on UHPLC–LTQ-IT-MS/MS datasets, (B) average antioxidant activity (2,2-diphenyl-1-picrylhydrazyl: DPPH) for seven *Acer* species plant extracts (different letters are indicative of statistically significant differences for observed bioactivities according to Duncan’s multiple-range test at *p* < 0.05), and (C) Heat map representation for the relative abundance of significantly discriminant metabolites based on the PLS-DA model (VIP > 0.7, *p* < 0.05). TR: *Acer triflorum*; PM: *Acer pictum* subsp. *mono*; BU: *Acer buergerianum*; KO: *Acer komarovii*; TA: *Acer tataricum*; PS: *Acer pseudosieboldianum*; PI: *Acer pictum*; PA: *Acer palmatum*.

Extracts obtained from seven different *Acer* species (Family: Aceraceae) were analyzed using UHPLC-LTQ-IT-MS/MS. A PLS-DA score plot indicated that the corresponding datasets for *Acer* species were clustered across PLS1 (16.0%) and PLS2 (17.4%), with model parameters R^2^X (0.449), R^2^Y (0.994), and Q^2^ (0.966), and *p*-value < 0.05 (Fig 2A). According to the PLS-DA score plot, the datasets for seven *Acer* species were clustered into three main groups. One group consisted of *Acer triflorum* (TR), *Acer buergerianum* (BU), *Acer komarovii* (KO), and *Acer pseudosieboldianum* (PS). The second group included *Acer pictum* subsp. *mono* (PM) and *Acer pictum* (PI), whereas the third group was represented by a single species, *Acer tataricum* (TA). On the basis of the PLS-DA model, 33 metabolites were selected as significantly discriminant among the seven different *Acer* species at a variable importance in the projection (VIP) value > 0.7 and *p*-value < 0.05. These metabolites were tentatively identified using measured mass spectra (m/z), retention times, mass fragment patterns, UV absorbance, elemental compositions, and i-FIT (theoretical isotope distribution) derived from the UHPLC-LTQ-IT-MS/MS and UPLC-Q-TOF-MS datasets, considering standard compounds, an in-house library, and published references (Table 2). Among the 33 significantly discriminant metabolites, we identified quinic acid derivatives (3), phenolic acid derivatives (7), flavonoid derivatives (14), miscellaneous (6), and non-identified (3) metabolites. A comparative analysis of the *Acer* species plant extracts based on DPPH (Fig 2B), ABTS, and FRAP (S1 Fig) assays indicated that TA had relatively higher antioxidant activities.

**Table 2:** Differences in the metabolites of seven *Acer* species identified using UHPLC-LTQ-IT-MS/MS and UPLC-Q-TOF-MS.

No.	Tentative Metabolites^a^	UHPLC-LTQ-IT-MS/MS				UPLC-Q-TOF-MS		ID^e^
		RT^b^(min)	[M—H]^-^	MS^n^ Fragment Pattern^c^	λ_max_	Elemental composition	i-FIT^d^ (norm)	
*Quinic acids*								
1	Quinic acid	0.95	191	173, 127, 111	240, 269	C7H11O6	0.004	LIB
2	Caffeoylquinic acid	1.52	353	191	231, 288	C16H17O9	0.320	LIB
3	Galloylquinic acid derivative	6.67	505	343 > 191, 169 > 125	216, 275	C23H21O13	0.766	-
*Flavanols (Tannins)*								
4	Maplexin B	1.23	315	-	218, 273	C13H15O9	0.368	-
5	Trigalloyl glucose	1.72	635	465 > 313	275	C27H23O18	-	Ref[31]
6	Ethyl gallate	6.60	197	169	226, 272	C9H9O5	0.030	Ref[32]
7	Ellagic acid	7.50	301	185	232, 255	C14H5O8	0.187	LIB
8	Ethyl gallate derivative	8.98	349	197 > 169	201, 280	C16H13O9	0.727	-
9	Maplexin E	7.40	619	467	231, 263	C27H23O17	0.079	LIB
10	Maplexin J	8.00	771	619 > 467	224, 270	C34H27O21	0.399	LIB
*Flavonols (Quercetins)*								
11	Quercetin-O-glucoside	7.60	463	301	219, 277	C21H19O12	1.652	LIB
12	Quercetin-(galloyl)glucoside	7.61	615	301	216, 279	C28H23O16	2.000	Ref[33]
13	Quercetin-O-arabinoside	7.99	433	301	230, 265	C20H17O11	0.578	Ref[34]
14	Quercetin-O-rhamnoside	8.09	447	301	244, 262	C21H19O11	0.635	Ref[34]
15	Quercetin-(galloyl)arabinoside	8.31	585	301	215, 273	C27H21O15	0.517	LIB
16	Quercetin-(galloyl)rhamnoside	9.03	599	301	214, 272	C28H23O15	0.382	Ref[33]
17	Quercetin-(digalloyl)rhamnoside	9.36	751	599 > 301	207, 270	C35H27O19	1.951	Ref[35]
18	Quercetin	9.74	301	-	206, 271	C15H9O7	0.212	LIB
*Flavonols (Kaempferols)*								
19	Kaempferol-O-glucoside	8.06	447	285	212, 267	C21H19O11	1.072	Ref[34]
20	Kaempferol-O-arabinoside	8.34	417	285	201, 267	C20H17O10	0.135	LIB
21	Kaempferol-(digalloyl)glucoside	8.64	751	599, 465 > 285	201, 267	C35H27O19	3.908	LIB
22	Kaempferol-O-rhamnoside	8.68	431	285	217, 275	C21H19O10	2.965	Ref[33]
23	Kaempferol-(galloyl)arabinoside	8.72	569	523 > 285	202, 273	C27H21O14	0.026	LIB
24	Kaempferol-(galloyl)rhamnoside	9.49	583	285	207, 270	C28H23O14	0.674	Ref[33]
Miscellane*ous*								
25	Procyanidin dimer	1.60	577	425 > 407	240, 269	C30H25O12	0.068	Ref[36]
26	Fraxin	1.60	369	207 > 192	240, 269	C16H17O10	0.142	Ref[37]
27	Aceroside VIII	9.01	593	293, 299 > 233, 191	270	C30H41O12	0.156	LIB
28	Aceroside III	9.47	591	293, 297 > 233, 191	278	C30H39O12	0.103	LIB
29	Trihydroxy-octadecadienoic acid	10.36	327	309, 291	214, 272	C18H31O5	0.750	Ref[38]
30	Acerogenin A	12.11	297	203	223, 278	C19H21O3	0.410	LIB
*Non-Identified*								
31	N.I (1)	0.95	229	191	234, 270	C12H21O4	0.495	-
32	N.I (2)	8.09	483	447	244, 262	C21H23O13	1.140	-
33	N.I (3)	9.22	603	451 > 341	212	C31H23O13	2.440	-

^a^ Tentative metabolites based on variable important projection (VIP) analysis with a cutoff value of 0.7 and *p*-value < 0.05.

^b^Retention time.

^c^ MS^n^ fragment patterns detected in the negative ion mode.

^d^ i-Fit is a measure of how well the observed isotope pattern matches the predicted isotope pattern for the formula on that line.

^e^ Identification: LIB, in house Library; Ref., reference

### Correlations between the discriminant metabolites and antioxidant activity in seven *Acer* species

The relative levels of significantly discriminant metabolites among the seven *Acer* species plant extracts were visualized using heat map representation (Fig 2C). Intriguingly, the levels of quercetin and its derivatives were relatively higher in PI, whereas quinic acid and its derivatives were abundant metabolites in PS. Further, acerogenin and acerogenin glycosides (aceroside) were selectively higher in TR, whereas those of tannin derivatives were higher in TA extracts compared with those of other *Acer* species.

**Fig 3:**
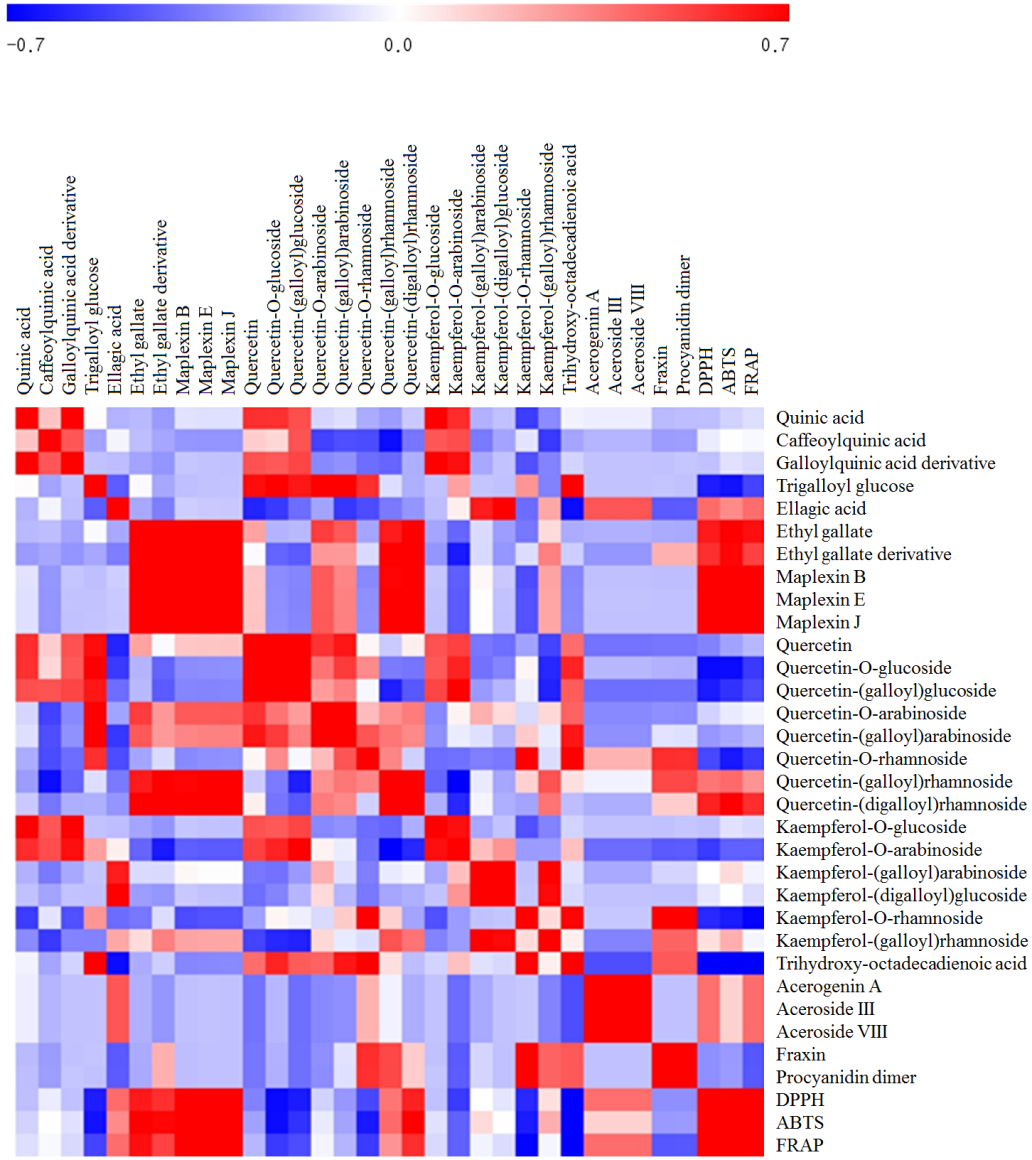
Correlation map representation of the relative levels of significantly different metabolites and observed bioactivities [2,2′-azino-bis (3-ethylbenzothiazoline-6-sulfonic acid), 2,2-diphenyl-1-picrylhydrazyl, and ferric reducing ability of plasma: ABTS, DPPH, and FRAP, respectively]. Each square indicates *r*, which is the Pearson correlation coefficient of a pair of metabolites and assayed activities. The red color indicates a positive (0 < *r* < 0.7) correlation and the blue color indicates a negative (-0.7 < *r* < 0) correlation.

Correlation analysis was performed to evaluate the individual contribution of metabolites toward the observed antioxidant activities (Fig 3). Accordingly, six tannin derivatives (ellagic acid, ethyl gallate and derivative, maplexin B, maplexin E, and maplexin J) exhibited a high positive correlation with antioxidant activity. Further, two quercetin derivatives (quercetin-(galloyl)rhamnoside and quercetin-(digalloyl)rhamnoside) and three acerogenin derivatives (acergenin, aceroside III, and aceroside VIII) showed positive but relatively weak correlations with antioxidant phenotypes.

### Bioconversion affects metabolite profiles and antioxidant activities in Aceraceae family plant extracts

**Fig 4:**
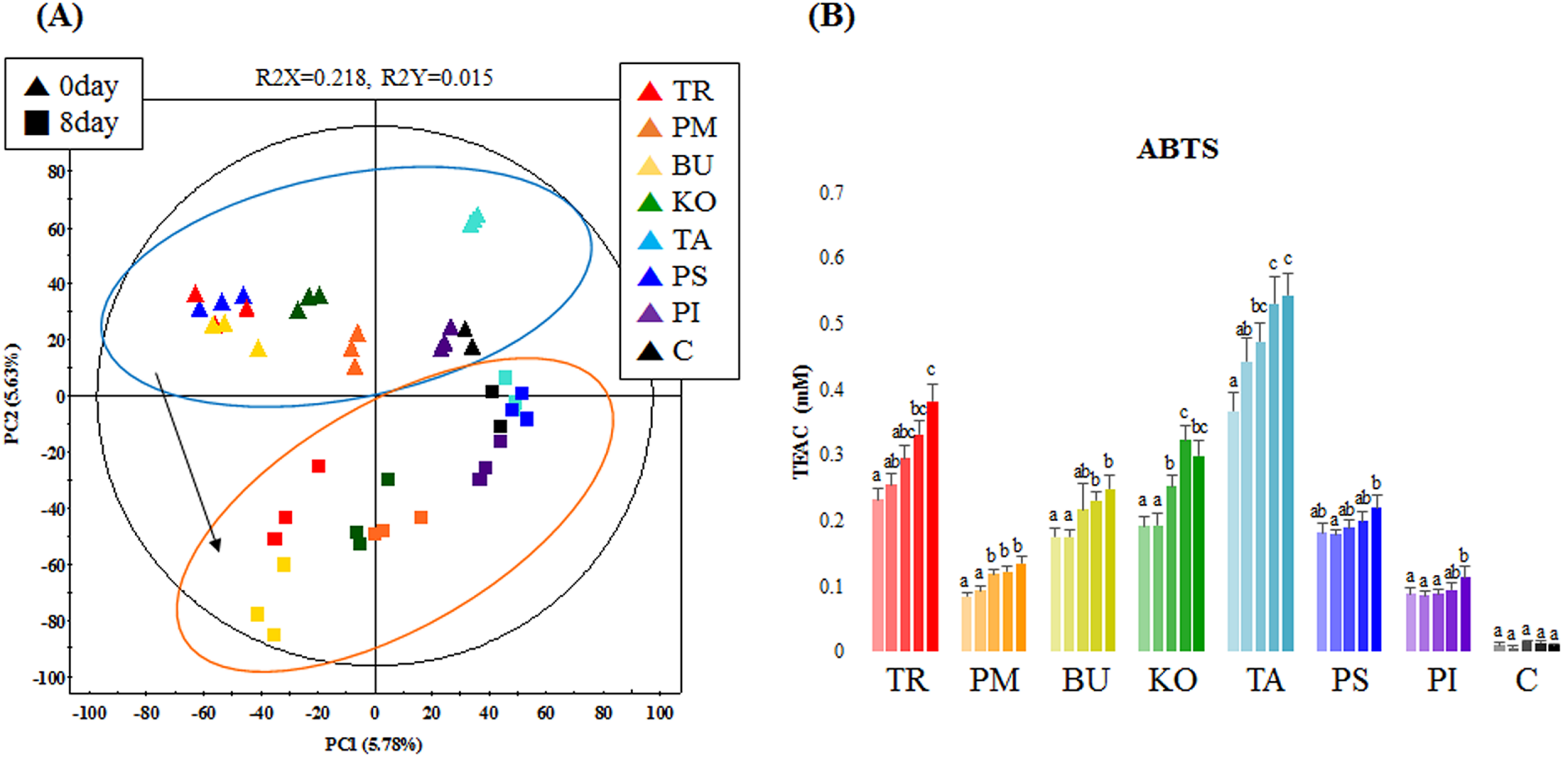
(A) Principal component analysis score plot derived from UHPLC-LTQ-IT-MS/MS datasets displaying a variance between unfermented (0 day) and fermented (8 days) samples, and (B) antioxidant activity assays, where each of the five columns indicates average 2,2′-azino-bis (3-ethylbenzothiazoline-6-sulfonic acid) (ABTS) activity for 0-, 2-, 4-, 6-, and 8-day fermented *Acer* extracts. TR: *Acer triflorum*; PM: *Acer pictum* subsp. *mono*; BU: *Acer buergerianum*; KO: *Acer komarovii*; TA: *Acer tataricum*; PS: *Acer pseudosieboldianum*; PI: *Acer pictum*; PA: *Acer palmatum*; C: control (only broth).

Following *A*. *oryzae*-mediated bioconversion of Aceraceae family plant extracts for 8 days, a time-correlated PCA was performed to evaluate the alterations in secondary metabolite profiles based on the UHPLC-LTQ-IT-MS/MS datasets. The PCA score plot showed variances of 6.24% and 5.94% along principal components PC1 and PC2, respectively (Fig 4A). The datasets for 0-day (▲) and 8-day (■) fermented extracts at the end of the bioconversion process were clearly separated along PC1, with each biological replicate representing a species clustered together. The discriminant metabolites that were significantly altered during the bioconversion process were selected at VIP value > 0.7 and *p*-value < 0.05 based on the corresponding PLS-DA model. A total of 36 metabolites were selected and divided into the following sub-groups: phenolic acid derivatives (12), flavonoid derivatives (12), miscellaneous (5), and non-identified (7) metabolites (Table 3). Considering the bioactivity phenotypes, we observed a concomitant increase in relative antioxidant activities for all *Acer*-extracts following the bioconversion process (Fig 4B). At 8 days after bioconversion, the order of the observed antioxidant activity was as follows: TA > TR > KO > BU > PS > PM > PI.

**Table 3:** Differential metabolites identified using UHPLC-LTQ-IT-MS/MS and UPLC-Q-TOF-MS in before/after bioconversion of *Acer*.

No.	Tentative Metabolites^a^	UHPLC-LTQ-IT-MS/MS				UPLC-Q-TOF-MS		ID^e^
		RT^b^(min)	[M—H]^-^	MS^n^ Fragment Pattern^c^	λ_max_	Elemental composition	i-FIT^d^ (norm)	
*Phenolic acids*								
1	Gallic acid	1.74	169	125 > 81	277	C7H5O5	n/a	LIB
2	Dihydroxybenzoic acid	2.29	153	109, 66	295	C7H5O4	0.269	Ref[39]
*Flavanols (Tannins)*								
3	Tetragalloyl glucose	7.61	787	635, 617 > 465	267, 351	C34H27O22	2.325	Ref[34]
4	Maplexin D	7.61	467	315, 297 > 169, 125	275	C20H19O13	0.754	LIB
5	Acalyphidin M1	7.85	907	755, 633, 435, 301	257, 347	C40H27O25	3.535	LIB
6	Maplexin E	7.95	619	467, 449 > 315, 297 > 169	266, 366	C27H23O17	1.070	LIB
7	Digalloyl-hexosyl-ellagic acid	8.14	765	301 > 257, 229, 185	254, 366	C35H25O20	4.065	Ref[40]
8	Valoneic acid dilactone	8.15	469	392 > 301, 169	254, 366	C21H9O13	-	Ref[40]
9	Ellagic acid	8.29	301	257, 229, 185	254, 366	C14H5O8	0.187	LIB
10	Ellagic acid derivative	8.54	749	301, 257, 229, 185	255, 355	-	-	-
11	Ellagic acid acetylpentoside	8.59	475	453 > 301, 169	255, 349	C21H15O13	-	Ref[41]
12	Ellagic acid derivative(2)	8.66	484	461 > 439, 301, 169	264, 347	-	-	-
*Flavonols*								
13	Kaempferol-O-(dirhamnosyl)hexoside	7.62	739	575, 284	269, 345	C33H39O19	3.188	Ref[42]
14	Rutin	7.81	609	301 > 179, 151	266, 350	C27H29O16	1.324	Ref[33]
15	Quercetin-O-glucoside	8.03	463	301 > 179, 151	254, 366	C21H19O12	0.285	LIB
16	Quercetin-(galloyl)glucoside	8.06	615	301 > 179, 151	257, 366	C28H23O16	1.388	Ref[33]
17	Quercetin derivative	8.24	917	615 > 301 > 179, 151	256, 349	C41H26O25	-	-
18	Quercetin-O-arabinoside	8.54	433	301 > 179, 151	263, 348	C20H18O11	0.578	Ref[34]
19	Kaempferol-O-glucoside	8.58	447	285 > 257, 229	256, 350	C21H19O11	0.125	Ref[34]
20	Quercetin-(galloyl)arabinoside	8.73	585	301 > 179, 151	266, 343	C27H21O15	2.265	LIB
21	Quercetin-O-rhamnoside	8.85	447	301 > 179, 151	255, 348	C21H19O11	1.692	Ref[34]
22	Isorhamnetin-O-rhamnoside	9.18	461	314	265, 339	C22H21O11	0.927	Ref[38]
23	Kaempferol-O-rhamnoside	9.38	431	285	265, 337	C21H19O10	-	Ref[33]
24	Quercetin	10.31	301	179, 151	347(sh)	C15H9O7	0.212	LIB
*Miscellaneous*								
25	Caffeoylquinic acid	2.07	353	309, 265 > 221, 191, 177	278	C16H17O9	0.407	LIB
26	Phloretin derivatives	8.65	581	417, 387, 357, 315, 297	255, 349	C27H343O14	1.712	-
27	Aceroside VIII	9.52	593	299 > 191, 177	265, 346	C30H41O12	1.016	LIB
28	Trihydroxy-octadecadienoic acid	11.02	327	291, 229, 211, 171	215, 285(sh)	C18H31O5	0.750	Ref[38]
29	Acerogenin A	12.60	297	203, 191	218	C19H21O3	0.188	LIB
*Non-Identified*								
30	N.I (1)	7.32	475	453, 429 > 265, 163	254, 366	-	-	-
31	N.I (2)	8.25	449	269	256, 348	-	-	-
32	N.I (3)	8.36	565	519	255, 352	-	-	-
33	N.I (4)	8.44	521	475, 444, 392, 358, 323, 301	255, 350	C26H33O11	3.385	-
34	N.I (5)	9.70	567	413, 293	-	-	-	-
35	N.I (6)	10.32	313	163, 149	219	C19H21O4	0.326	-
36	N.I (7)	12.16	299	191, 177, 121	218, 284(sh)	C19H23O3	0.018	-

^a^ Tentative metabolites based on variable important projection (VIP) analysis with a cutoff value of 0.7 and *p*-value < 0.05.

^b^ Retention time.

^c^ MS^n^ fragment patterns detected in the negative ion mode.

^d^ i-Fit is a measure of how well the observed isotope pattern matches the predicted isotope pattern for the formula on that line.

^e^ Identification: LIB, in house Library; Ref., references.

**Fig 5:**
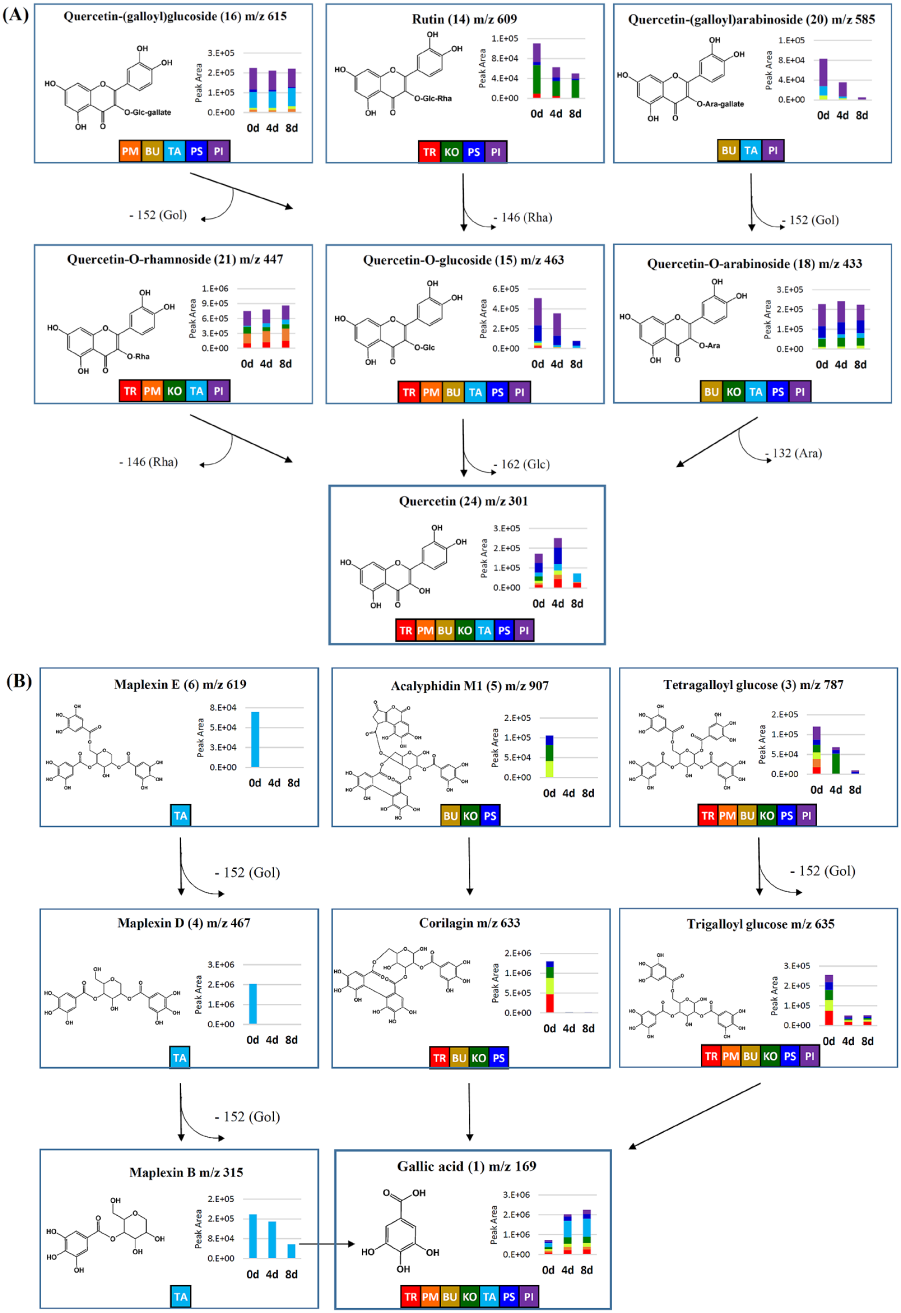
Proposed bioconversion pathways derived for the selected metabolites in UHPLC-LTQ-IT-MS/MS datasets relevant to the time-correlated production of (A) quercetin, and (B) gallic acid during the fermentative bioconversion process. The inset graphs indicate the metabolite peak area plotted along the y-axis, whereas the incubation period (0, 4, and 8 days) during fermentation is plotted along the x-axis. The seven *Acer* species are indicated with different color codes in the inset graph.

Further, we visualized variations in the relative levels and bioconversion mechanisms of selected sets of discriminant metabolites, including quercetin and tannin derivatives (Fig 5). As shown in Fig 5A, the relative levels of rutin, quercetin-(galloyl)arabinoside, and quercetin-*O*-glucoside were considerably decreased during the bioconversion process, whereas the remaining metabolites showed marginal changes. Intriguingly, the quercetin levels were steadily elevated from 0 to 4 days and decreased thereafter until the end of the bioconversion process. Similarly, a chain of bioconversion events involving the conversion of tannin derivatives (maplexin E, acalyphidin M1, tetragalloyl glucose, maplexin D, corilagin, trigalloyl glucose, and maplexin B) to gallic acid might have contributed to its elevated levels at the end of bioprocessing (Fig 5B). Notably, following bioconversion, the gallic acid levels were significantly higher in TA compared with the other *Acer* species.

## Discussion

Throughout human history, plant-derived natural products and metabolic intermediates have served as dietary supplements, nutraceuticals, and pharmacological agents, and more recently have been used as templates for drug discovery. In this study, we mainly employed metabolic profiling, bioactivity screening, and subsequent bioconversion to examine the antioxidant bioactivities of extracts derived from members of three regionally abundant plant families: Aceraceae, Asteraceae, and Rosaceae. In the present study we focused on altered antioxidant activities mainly as the higher plants and their constituents provide a rich source of natural antioxidants with umpteen pharmaceutical, health, and cosmetic applications. Especially, the natural phytochemicals in plants have been receiving increased interest from consumers for their beneficial health effects owing to their antioxidant metabolites. The bioconversion of plant natural products coupled with microbial production of bioactive compounds from renewable plant resources is recognized as a sustainable alternative to synthetic processes. Hence, fermentative bioprocesses used for the bioconversion of plant materials are considered advantageous owing to their low energy requirements, low waste emissions, and efficient recovery of end products [20].

On the basis of the initial screening studies that revealed higher antioxidant activities in Aceraceae family plant extracts, we selectively examined the metabolic profiles of *Acer* species extracts and evaluated the correlations between the significantly discriminant metabolites and related antioxidant activities. Metabolic profiling followed by multivariate statistical analysis indicated that UHPLC-LTQ-IT-MS/MS datasets for seven *Acer* species extracts were separated into three groups (Fig 2A), which showed marked metabolic disparity, pertaining to the flavonoid and tannin contents, among the *Acer* family plant extracts. Hence, we observed a marked variation in the levels of antioxidant activities, with *Acer tataricum* (TA) showing the highest relative levels of antioxidant metabolites (Fig 2B and 2C). Substantiating our results, interspecies variations in secondary metabolite compositions as well as related bioactivities have previously been described for plant species, including those in the genus *Acer* [7, 21]. Further, correlation analysis indicating the contribution of individual metabolites to bioactivities revealed that most tannins, abundant in TA, showed a strong positive correlation with antioxidant activity (Fig 3). Moreover, we conjecture that while the bioconversion process, there would be a large spectrum of metabolites that either remained undetected, uncharacterized, transiently produced, or not selected as discriminant at VIP>0.7 and *p*<0.5 from PLS-DA model, which might have contributed to the observed antioxidant activities. Further, it appears hard to conceive the perfect one-to-one relationship between metabolites and associated bioactivity, because the fermentation environment is extremely dynamic and has an overwhelming range of byproduct including the antioxidant phenolic compounds influencing the phenotypes. Overall, these results indicated that the observed metabolic disparity among the Aceraceae family extracts contribute to their different bioactivities. In congruence to our study, Choudhary and Swarnkar [22] have previously reported that a disparity in phenolic and flavonoid contents results in different antioxidant potentials among extracts from the same plant species.

The extensive and safe application of *A*. *oryzae* in fermentative bioprocesses owing to its abundant secretion of hydrolytic enzymes (α-galactosidase, β-glucosidase, xylosidase, and tannase) provide justification for its application in *Acer* extract fermentation for valuable chemicals. Following *A*. *oryzae*-mediated bioconversion, the metabolite compositions of the *Acer* family plant extracts changed considerably, thereby affecting the associated antioxidant activities. As indicated in the PCA score plot shown in Fig 4A, a clear metabolic disparity was evident, discriminating the 8-day fermented samples from the unfermented 0-day metabolic profiles. The tentative mechanism for the bioconversion pathway based on UHPLC-LTQ-IT-MS/MS analysis indicates that the relative levels of quercetin derivatives steadily decreased, whereas those of end-product quercetin increased, which can be attributed to the hydrolytic action of β-glucosidase enzymes breaking glucosidic bonds. For example, β-glucosidases from *A*. *oryzae* can hydrolyze the glucosidic bonds of piceid to release free resveratrol compounds [23]. Hence, we assume that *A*. *oryzae*-released enzymes convert rutin, quercetin-*O*-glucoside, and quercetin-*O*-arabinoside into the corresponding deglycoside compounds, and finally into end-product quercetin aglycones. Interestingly, the relative abundance of quercetin was increased up to 4 days and then decreased until the end of bioconversion process. We conjecture that the observed reduction in quercetin levels during the latter half of the bioconversion process could be associated with the release of quercetinases in the fermentation matrix. Quercetinases are extracellular enzymes produced by several *Aspergillus* species that promote the enzymatic oxygenation and cleavage of quercetin [24]. Another noteworthy point relates to gallic acid production following the successive bioconversion steps degrading tannin derivatives (Fig 5B). The reaction mechanism involves the loss of galloyl moieties from highly polymerized tannins, e.g., maplexin E, corilagin, and tetragalloyl glucose, converting these into smaller residues, mainly gallic acid. Hence, the successive bioconversion of precursor compounds and stable accumulation of gallic acid results in its higher abundance at the end of the bioconversion process. Tannase enzymes from *A*. *oryzae* have been reported to specifically catalyze the cleavage of a galloyl moiety from tannins, resulting in the release of gallic acid and glucose moieties [25]. By analogy, the aforementioned mechanisms are similar to those reported for tannic acid conversion to gallic acid mediated by *A*. *awamori*-secreted tannases [26]. Hence, the relatively higher abundance of gallic acid in TA end products compared with other *Acer* species might be affected by the relatively higher abundance of gallic acid precursor compounds, e.g., maplexin B, D, and E.

We accordingly propose that the observed trends of increasing antioxidant bioactivities during the bioconversion process for all *Acer* species plant extracts could be correlated with the enhanced release and stable accumulation of secondary metabolites with higher antioxidant potentials (Fig 4B). In particular, the initial titers of flavonoid glycosides and hydrolysable tannins were converted into quercetin and gallic acid derivatives with relatively higher antioxidant potentials compared with the precursors. Our assumptions are in agreement with previous studies describing the higher antioxidant bioactivities of aglycones or free-moiety compounds [27, 28]. Further, similar studies employing metabolomic approaches have confirmed enhanced bioactivities following the bioconversion process for green tea [29] and soybean [30] extracts.

## Conclusions

In the present study, we adopted an integrated metabolomic approach involving MS-based analysis and bioactivity screening of various plant extracts followed by systematic delineation of their fermentative bioconversion to high value metabolites. The *A*. *oryzae*-mediated bioconversion of *Acer* species plant extracts revealed that the precursor metabolites including various flavanols (tannins) and flavonols (quercetin precursors) were converted to relatively stable derivatives, such as gallic acid and quercetins, respectively. Hence, a relatively stable accumulation of end-product derivative might affect the higher bioactivities of plant metabolite extracts following a bioconversion process. The methodology adopted in this work can accordingly serve as a platform toward enhanced recovery and economic production of plant derived natural products having high commercial value. However, in this regard, we propose that further detailed studies involving process optimization, pharmacological efficacies, and toxicity assessment of plant extract bioconversion products are needed.

## Supporting information

S1 Fig Antioxidant activity test, ABTS (A) and FRAP (B) for the seven *Acer* species. Different letters are significantly different according to Duncan’s multiple-range test (*p* < 0.05).
(PDF)

S2 Fig PLS-DA score plot of each *Acer* species derived from UHPLC-LTQ-IT-MS/MS datasets displaying variance between unfermented (0- day), 4- days and 8- days fermented. Here, TR, *Acer triflorum*; PM, *Acer pictum* subsp.*mono*; BU, *Acer buergerianum*; KO, *Acer komarovii*; TA, *Acer tataricum*; PS, *Acer pseudosieboldianum*; PI, *Acer pictum*; PA, *Acer palmatum*.
(PDF)

S1 Table Peak area and ANOVA of each significantly different metabolites replicates. (XLSX)

S2 Table Anonymized data set afterward alignment. (XLSX)

## References

[1] Martins S, Mussatto SI, Martínez-Avila G, Montañez-Saenz J, Aguilar CN, Teixeira JA. Bioactive phenolic compounds: production and extraction by solid-state fermentation. A review. Biotechnol Adv. 2011; 29: 365–373. doi: 10.1016/j.biotechadv.2011.01.008 21291993 10.1016/j.biotechadv.2011.01.008

[2] Kawser Hossain M, Abdal Dayem A., Han J, Yin Y, Kim K, Kumar Saha S, et al Molecular mechanisms of the anti-obesity and anti-diabetic properties of flavonoids. Int J Mol Sci. 2016; 17: 569 doi: 10.3390/ijms17040569 27092490 10.3390/ijms17040569 PMC4849025

[3] Hasani-Ranjbar S, Nayebi N, Larijani B, Abdollahi M. A systematic review of the efficacy and safety of herbal medicines used in the treatment of obesity. World J Gastroenterol. 2009; 15: 3073 doi: 10.3748/wjg.15.3073 19575486 10.3748/wjg.15.3073 PMC2705729

[4] Zheng Z, Shetty K. Solid-state bioconversion of phenolics from cranberry pomace and role of *Lentinus edodes* β-glucosidase. J Agric Food Chem. 2000; 48: 895–900. 10725170 10.1021/jf990972u

[5] Gallage NJ, Møller BL. Vanillin–bioconversion and bioengineering of the most popular plant flavor and its *de novo* biosynthesis in the vanilla orchid. Mol plant, 2015; 8: 40–57. doi: 10.1016/j.molp.2014.11.008 25578271 10.1016/j.molp.2014.11.008

[6] van Gelderen DM, De Jong PC, Oterdoom HJ. Maples of the World. Portland: Timber Press, 1994.

[7] Bi W, Gao Y, Shen J, He C, Liu H, Peng Y, et al Traditional uses, phytochemistry, and pharmacology of the genus *Acer* (maple): a review. J ethnopharmacol. 2016; 189: 31–60. 27132717 10.1016/j.jep.2016.04.021

[8] Bessada SM, Barreira JC, Oliveira MBP. Asteraceae species with most prominent bioactivity and their potential applications: A review. Ind Crops Prod. 2015; 76: 604–615.

[9] Wu QX, Shi YP, Jia ZJ. Eudesmane sesquiterpenoids from the Asteraceae family. Nat Prod Rep. 2006; 23: 699–734. doi: 10.1039/b606168k 17003906 10.1039/b606168k

[10] Son SY, Kim NK, Lee S, Singh D, Kim GR, Lee JS, et al Metabolite fingerprinting, pathway analyses, and bioactivity correlations for plant species belonging to the Cornaceae, Fabaceae, and Rosaceae families. Plant Cell Rep. 2016; 35: 1917–1931. doi: 10.1007/s00299-016-2006-y 27344340 10.1007/s00299-016-2006-y

[11] Hur SJ, Lee SY, Kim YC, Choi I, Kim GB. Effect of fermentation on the antioxidant activity in plant-based foods. Food Chem. 2014; 160: 346–356. doi: 10.1016/j.foodchem.2014.03.112 24799248 10.1016/j.foodchem.2014.03.112

[12] Suh DH, Jung ES, Park HM, Kim SH, Lee S, Jo YH, et al Comparison of metabolites variation and antiobesity effects of fermented versus nonfermented mixtures of *Cudrania tricuspidata*, *Lonicera caerulea*, and soybean according to fermentation in vitro and in vivo. PloS one. 2016; 11: e0149022 doi: 10.1371/journal.pone.0149022 26848749 10.1371/journal.pone.0149022 PMC4743955

[13] Lee M, Cho JY, Lee YG, Lee HJ, Lim SI, Park SL, et al Bioconversion of Capsaicin by *Aspergillus oryzae*. J Agric Food Chem. 2015; 63: 6102–6108. doi: 10.1021/acs.jafc.5b01730 26072923 10.1021/acs.jafc.5b01730

[14] Huynh NT, Smagghe G, Gonzales GB, Van Camp J, Raes K. Extraction and bioconversion of kaempferol metabolites from cauliflower outer leaves through fungal fermentation. Biochem Eng J. 2016; 116: 27–33.

[15] Lee S, Seo MH, Oh DK, Lee CH. Targeted metabolomics for *Aspergillus oryzae*-mediated biotransformation of soybean isoflavones, showing variations in primary metabolites. Biosci Biotechnol Biochem. 2014; 78: 167–174. doi: 10.1080/09168451.2014.877827 25036500 10.1080/09168451.2014.877827

[16] Li H, Wang Z. Comparison in antioxidant and antitumor activities of pine polyphenols and its seven biotransformation extracts by fungi. PeerJ. 2017; 5: e3264 doi: 10.7717/peerj.3264 28560092 10.7717/peerj.3264 PMC5444373

[17] Re R, Pellegrini N, Proteggente A, Pannala A, Yang M, Rice-Evans C. Antioxidant activity applying an improved ABTS radical cation decolorization assay. Free Radic Biol Med. 1999; 26: 1231–1237. 10381194 10.1016/s0891-5849(98)00315-3

[18] Dietz BM, Kang YH, Liu G, Eggler AL, Yao P, Chadwick LR, et al Xanthohumol isolated from *Humulus lupulus* inhibits menadione-induced DNA damage through induction of quinone reductase. Chem Res Toxicol. 2005; 18: 1296–1305. doi: 10.1021/tx050058x 16097803 10.1021/tx050058x PMC7395304

[19] Benzie IF, Strain JJ. The ferric reducing ability of plasma (FRAP) as a measure of “antioxidant power”: the FRAP assay. Anal Biochem. 1996; 239: 70–76. doi: 10.1006/abio.1996.0292 8660627 10.1006/abio.1996.0292

[20] Zhou J, Du G, Chen J. Novel fermentation processes for manufacturing plant natural products. Curr Opin Biotechnol. 2014; 25: 17–23. doi: 10.1016/j.copbio.2013.08.009 24484876 10.1016/j.copbio.2013.08.009

[21] Tohma H, Köksal E, Kılıç Ö, Alan Y, Yılmaz MA, Gülçin I, et al RP-HPLC/MS/MS analysis of the phenolic compounds, antioxidant and antimicrobial activities of *Salvia* L. species. Antioxidants. 2016; 5: 38. 10.3390/antiox5040038 PMC5187536 27775656

[22] Choudhary RK, Swarnkar PL. Antioxidant activity of phenolic and flavonoid compounds in some medicinal plants of India. Nat Prod Res. 2011; 25: 1101–1109. doi: 10.1080/14786419.2010.498372 21726132 10.1080/14786419.2010.498372

[23] Zhang J C, Li D, Yu H, Zhang B, Jin F. Purification and characterization of piceid-β-D-glucosidase from *Aspergillus oryzae*. Process Biochem. 2007; 42: 83–88.

[24] Schaab MR, Barney BM, Francisco WA. Kinetic and spectroscopic studies on the quercetin 2, 3-dioxygenase from *Bacillus subtilis*. Biochemistry. 2006; 45: 1009–1016. doi: 10.1021/bi051571c 16411777 10.1021/bi051571c

[25] Abdel-Nabey MA, Sherief AA, El-Tanash AB. Tannin biodegradation and some factors affecting tannase production by two *Aspergillus* sp. Biotechnology. 2011; 10: 149–158.

[26] Rajak RC, Singh A, Banerjee R. Biotransformation of hydrolysable tannin to ellagic acid by tannase from *Aspergillus awamori*. Biocatal Biotransformation. 2017; 35: 27–34.

[27] Duenas M, Fernández D, Hernández T, Estrella I, Muñoz R. Bioactive phenolic compounds of cowpeas (*Vigna sinensis L*.) modifications by fermentation with natural microflora and with *Lactobacillus plantarum* ATCC 14917. J Sci Food Agric. 2005; 85: 297–304.

[28] Singh HB, Singh BN, Singh SP, Nautiyal CS. Solid-state cultivation of *Trichoderma harzianum* NBRI-1055 for modulating natural antioxidants in soybean seed matrix. Bioresour Technol. 2010; 101: 6444–6453. doi: 10.1016/j.biortech.2010.03.057 20363120 10.1016/j.biortech.2010.03.057

[29] Kim MJ, John KM, Choi JN, Lee S, Kim AJ, Kim YM, et al Changes in secondary metabolites of green tea during fermentation by *Aspergillus oryzae* and its effect on antioxidant potential. Food Res Int. 2013; 53: 670–677.

[30] John KM, Jung ES, Lee S, Kim JS, Lee CH. Primary and secondary metabolites variation of soybean contaminated with *Aspergillus sojae*. Food Res Int. 2013; 54: 487–494.

[31] Meyers KJ, Swiecki TJ, Mitchell AE. Understanding the native Californian diet: Identification of condensed and hydrolyzable tannins in tanoak acorns (*Lithocarpus densiflorus*). J Agric Food Chem. 2006; 54: 7686–7691. 17002440 10.1021/jf061264t

[32] He Z, Xia W. Analysis of phenolic compounds in Chinese olive (*Canarium album* L.) fruit by RPHPLC–DAD–ESI–MS. Food Chem. 2007; 105: 1307–1311.

[33] Gu D, Yang Y, Bakri M, Chen Q, Xin X, Aisa HA. A LC/QTOF–MS/MS Application to Investigate Chemical Compositions in a Fraction with Protein Tyrosine Phosphatase 1B Inhibitory Activity from *Rosa Rugosa* Flowers. Phytochem Anal. 2013; 24: 661–670. doi: 10.1002/pca.2451 23813906 10.1002/pca.2451

[34] de Brito ES, de Araújo MCP, Lin LZ, Harnly J. Determination of the flavonoid components of cashew apple (*Anacardium occidentale*) by LC-DAD-ESI/MS. Food Chem. 2007; 105: 1112–1118. doi: 10.1016/j.foodchem.2007.02.009 25544795 10.1016/j.foodchem.2007.02.009 PMC4276398

[35] Chan EWL, Gray AI, Igoli JO, Lee SM, Goh JK. Galloylated flavonol rhamnosides from the leaves of *Calliandra tergemina* with antibacterial activity against methicillin-resistant *Staphylococcus aureus* (MRSA). Phytochemistry. 2014; 107: 148–154. doi: 10.1016/j.phytochem.2014.07.028 25174555 10.1016/j.phytochem.2014.07.028

[36] Tomás-Barberán FA, Gil MI, Cremin P, Waterhouse AL, Hess-Pierce B, Kader AA. HPLC− DAD− ESIMS analysis of phenolic compounds in nectarines, peaches, and plums. J Agric Food Chem. 2001; 49: 4748–4760. 11600017 10.1021/jf0104681

[37] Cheng L, Zhang X, Zhang M, Zhang P, Song Z, Ma Z, et al Characterization of chemopreventive agents from the dichloromethane extract of *Eurycorymbus cavaleriei* by liquid chromatography–ion trap mass spectrometry. J Chromatogr A. 2009; 1216: 4859–4867. doi: 10.1016/j.chroma.2009.04.031 19439309 10.1016/j.chroma.2009.04.031

[38] Farag MA, Sakna ST, El-fiky NM, Shabana MM, Wessjohann LA. Phytochemical, antioxidant and antidiabetic evaluation of eight *Bauhinia* L. species from Egypt using UHPLC–PDA–qTOF-MS and chemometrics. Phytochemistry. 2015; 119: 41–50. doi: 10.1016/j.phytochem.2015.09.004 26410474 10.1016/j.phytochem.2015.09.004

[39] Gruz J, Novák O, Strnad M. Rapid analysis of phenolic acids in beverages by UPLC–MS/MS. Food Chem. 2008; 111: 789–794.

[40] Abu-Reidah IM, Ali-Shtayeh MS, Jamous RM, Arráez-Román D, Segura-Carretero A. HPLC–DAD–ESI-MS/MS screening of bioactive components from *Rhus coriaria* L.(Sumac) fruits. Food Chem. 2015; 166: 179–191. doi: 10.1016/j.foodchem.2014.06.011 25053044 10.1016/j.foodchem.2014.06.011

[41] Dall’Asta M, Calani L, Tedeschi M, Jechiu L, Brighenti F, Del Rio D. Identification of microbial metabolites derived from in vitro fecal fermentation of different polyphenolic food sources. Nutrition. 2012; 28: 197–203. doi: 10.1016/j.nut.2011.06.005 22208556 10.1016/j.nut.2011.06.005

[42] Truchado P, Vit P, Ferreres F, Tomas-Barberan F. Liquid chromatography–tandem mass spectrometry analysis allows the simultaneous characterization of C-glycosyl and O-glycosyl flavonoids in stingless bee honeys. J Chromatogr A. 2011; 1218: 7601–7607. doi: 10.1016/j.chroma.2011.07.049 21831383 10.1016/j.chroma.2011.07.049

