# Supplementary material for: Systematic metabolic profiling and bioactivity assays for bioconversion of Aceraceae family
Source: PLoS One. 2018 Jun 7;13(6):e0198739. doi: 10.1371/journal.pone.0198739 (PMC5991731; doi:10.1371/journal.pone.0198739)
Supplement: S1 Fig — Different letters are significantly different according to Duncan’s multiple-range test (p < 0.05). (PDF) [file pone.0198739.s001.pdf]

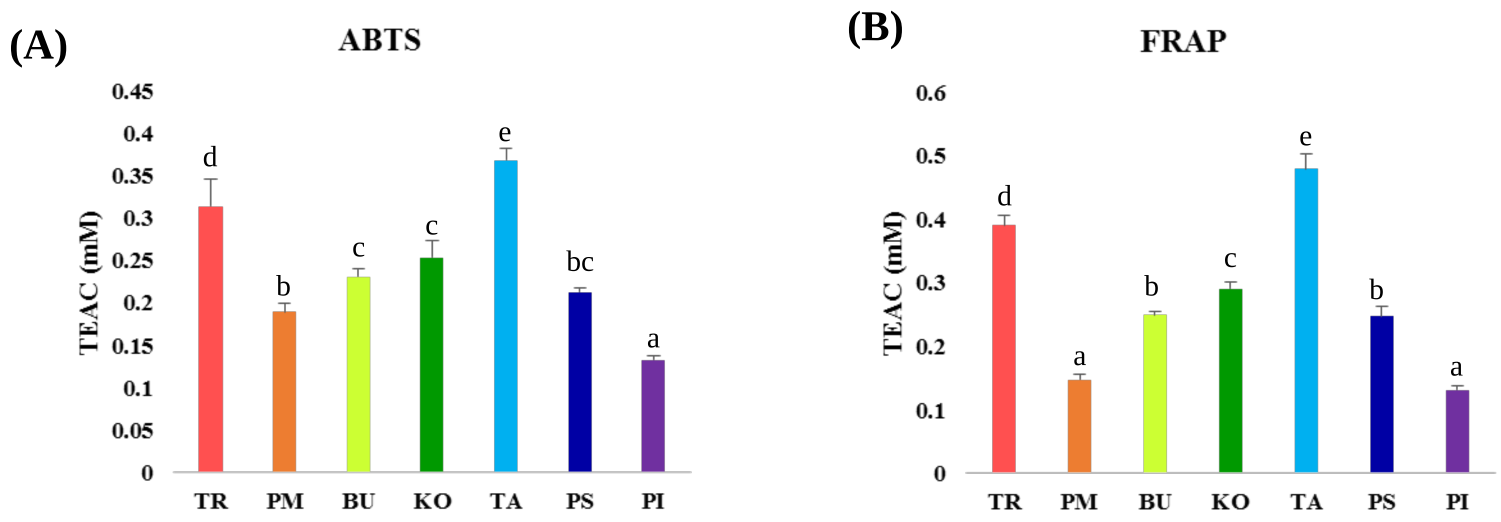

S1 Figure. Antioxidant activity test, ABTS (A) and FRAP (B) for the seven *Acer* species; Different letters are significantly different according to Duncan's multiple-range test ($p < 0.05$).
