# Supplementary material for: Systematic metabolic profiling and bioactivity assays for bioconversion of Aceraceae family
Source: PLoS One. 2018 Jun 7;13(6):e0198739. doi: 10.1371/journal.pone.0198739 (PMC5991731; doi:10.1371/journal.pone.0198739)
Supplement: S2 Fig — Here, TR, Acer triflorum; PM, Acer pictum subsp.mono; BU, Acer buergerianum; KO, Acer komarovii; TA, Acer tataricum; PS, Acer pseudosieboldianum; PI, Acer pictum; PA, Acer palmatum. (PDF) [file pone.0198739.s002.pdf]

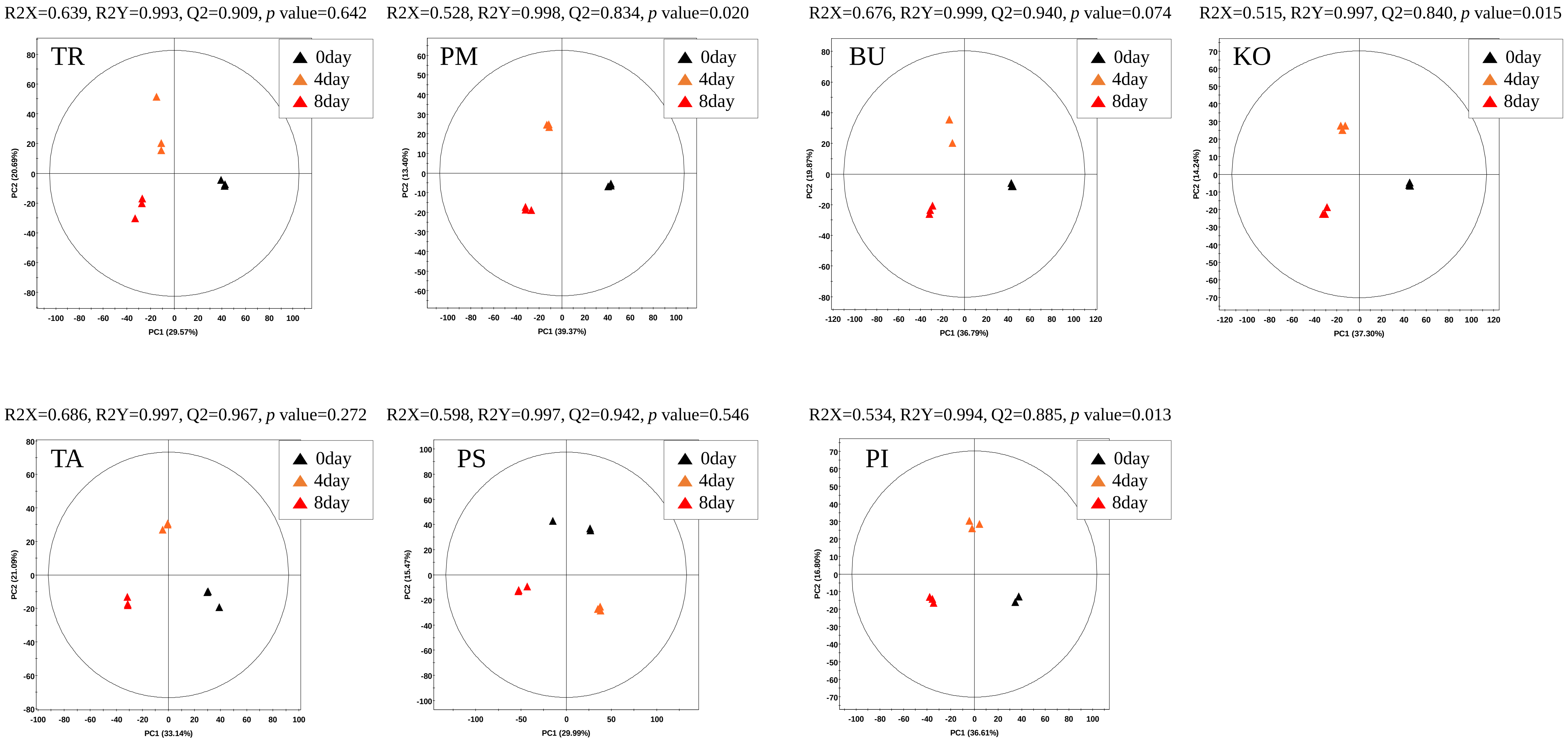

S2 Figure. PLS-DA score plot of each *Acer* species derived from UHPLC-LTQ-IT-MS/MS datasets displaying variance between unfermented (0- day), 4- days and 8- days fermented. Here, TR, *Acer triflorum*; PM, *Acer pictum* subsp.*mono*; BU, *Acer buergerianum*; KO, *Acer komarovii*; TA, *Acer tataricum*; PS, *Acer pseudosieboldianum*; PI, *Acer pictum*; PA, *Acer palmatum.*
